# Tracking by Risky Particle Filtering over Sensor Networks

**DOI:** 10.3390/s20113109

**Published:** 2020-05-31

**Authors:** Jaechan Lim, Hyung-Min Park

**Affiliations:** 1 Department of Electronic Engineering, Sogang University, Seoul 04107, Korea; jaechan@gmail.com; 2 Department of Electrical Engineering and Computer Science, University of Michigan, Ann Arbor, MI 48109, USA

**Keywords:** degeneracy, minimax, particle filtering, risk, received signal strength, target tracking, wireless sensor networks

## Abstract

The system of wireless sensor networks is high of interest due to a large number of demanded applications, such as the Internet of Things (IoT). The positioning of targets is one of crucial problems in wireless sensor networks. Particularly, in this paper, we propose minimax particle filtering (PF) for tracking a target in wireless sensor networks where multiple-RSS-measurements of received signal strength (RSS) are available at networked-sensors. The minimax PF adopts the maximum risk when computing the weights of particles, which results in the decreased variance of the weights and the immunity against the degeneracy problem of generic PF. Via the proposed approach, we can obtain improved tracking performance beyond the asymptotic-optimal performance of PF from a probabilistic perspective. We show the validity of the employed strategy in the applications of various PF variants, such as auxiliary-PF (APF), regularized-PF (RPF), Kullback–Leibler divergence-PF (KLDPF), and Gaussian-PF (GPF), besides the standard PF (SPF) in the problem of tracking a target in wireless sensor networks.

## 1. Introduction

Recently, research related to Internet of Things (IoT) is a hot area where many people from diverse fields are working due to the demand of smart systems that enable the ability to transfer data over a network without requiring human interactions by using smart devices, such as smartphones [1,2,3,4]. In this smart system, a sensor network is formed to interconnect densely populated sensors for data communications and processing among the sensors. A traditional field of wireless sensor networks contributes to enabling the IoT [5,6]. There are many applications that can be enabled by wireless sensor network-based IoT, such as health care systems, transportation, defense applications, and smart-home systems.

Depending on applications, a sensor network can comprise different types of sensors. Tracking a moving target is an important problem in wireless sensor networks where the position and/or velocity of a moving object within the region of interest is estimated. The problem can be classified by three main methods that can be employed for the tracking application according to the type of physical measurement read at sensors. The main types of measurements are the time delay of arrival (TDOA) [7,8], the direction of arrival (DOA) [9,10], and the received signal strength (RSS) [11,12,13,14,15,16,17,18,19] of the signal generated from the tracked target, respectively. RSS measurement of the sensors represents received energy or power emitted by the target. RSS-based sensors have advantages of simpler architecture and less expensive cost than the cases of TDOA and DOA. In this paper, we propose a tracking approach that uses multi-RSS measurements. In particular, we propose the minimax particle filtering (PF) approach for a target tracking, which estimates the trajectory and velocity.

Within the past few decades, since PF was proposed [20], PF has shown asymptotically optimal performance in various time-varying state estimation problems, particularly for nonlinear problems. Further, to advance the performance of the standard PF, various versions of PF have been proposed since its initial proposal [21,22,23,24,25,26,27,28,29,30]. Based on the theoretical background, PF can perform optimally by using an infinite number of particles in nonlinear problems. Nonetheless, from time to time, we cannot obtain ideal results via PF due to various unexpected reasons. One main reason is the degeneracy phenomenon, i.e., after a few time steps since the initialization of the algorithm, there is only one particle that has significant weight, whereas all the other particles have nearly zeros weights. Consequently, the variance of the weights of particles only increases over time, which results in insufficient performance due to the loss of particle diversity [31]. To overcome the degeneracy issue, the resampling [32,33] can be applied that regenerates high-quality particles duplicated. On the other hand, as a by-effect of the resampling, PF undergoes particle impoverishment that we have only identical particles over a short time, especially when the state noise is little. Therefore, maintaining a constantly small variance of the weights of particles is desired to avoid the unsatisfactory performance of PF. For this goal, we adopt the strategy of the minimax approach that incurs a significantly mitigated variance of the weights of particles and results in improved performance of PF. In this approach, we minimize the maximum risk function. That is, we adopt the maximum risk when computing the weights of particles; consequently, the proposed approach minimizes the highly increased risk function compared to the cases of regular PFs. In other words, we devalue the quality of a particle as low as possible.

In this paper, we propose minimax-PF (MPF) for the target tracking based on multi-RSS-measurements at up to ten sensors. The minimax PF approach was initially introduced in Reference [34] recently, where a maneuvering target is tracked based on the range and bearing, which are only two measurements; therefore, the methodology was not fully investigated in terms of the number of measurements, clarity of the derivation of the approach, analysis of risk and weight concerning to all compared variants of PFs. The investigation of the method in the application of networked sensor measurements where a large number of sensors are available is more appropriate for the verification of the approach. In Reference [35], the minimax strategy was adopted in an interactive-multiple-model algorithm implemented by PF with a couple of range and bearing measurements only. In this paper, we verify with up to ten RSS measurements in wireless sensor networks to generalize the validity of the proposed approach with extensive experimental results and analysis. The number of employed measurements is a key factor to assess and generalize the validity of the proposed minimax PF approach. We show the outperforming results of the proposed MPFs compared to the various regular PFs, i.e., standard PF, APF, GPF, RPF, and KLDPF.

For notations, matrices, vectors, and scalars are denoted by bold uppercase letters, bold lowercase letters, and lowercase letters, respectively, and ⊤ denotes the transpose of a matrix.

## 2. Problem Formulation

We track a target based on a number of RSS measurements in a two-dimensional space. The scenario also can be a localization problem, but tracking is necessary when we have limited time or computational complexity in positioning the target.

### 2.1. Dynamic State Model

The movement of the target is mainly driven by the state noise that is also the random acceleration of the target. The state and measurement variables are denoted by ψ and m, respectively, and the dynamic state function is described referring to Reference [36,37,38] as follows:

(1) $$\psi_{t}=D\psi_{t-1}+E\theta_{t},$$

where

(2) $$\begin{array}{ccc} \psi_{t} & = & {\left[\psi_{1,t},\psi_{2,t},\ldots ,\psi_{1,L}\right]}^{⊤} \end{array}$$

(3) $$\begin{array}{cc} = & {\left[r_{x,t},r_{y,t},v_{x,t},v_{y,t}\right]}^{⊤}, \end{array}$$

(4) $$\begin{array}{ccc} \psi_{t-1} & = & {\left[r_{x,t-1},r_{y,t-1},v_{x,t-1},v_{y,t-1}\right]}^{⊤}, \end{array}$$

(5) $$\begin{array}{ccc} \theta_{t} & = & {\left[\theta_{x,t},\theta_{y,t}\right]}^{⊤} \end{array}$$

(6) $$D=\left[\begin{array}{cccc} 1 & 0 & T_{s} & 0\\ 0 & 1 & 0 & T_{s}\\ 0 & 0 & 1 & 0\\ 0 & 0 & 0 & 1 \end{array}\right],\;\;E=\left[\begin{array}{cc} \frac{T_{s}^{2}}{2} & 0\\ 0 & \frac{T_{s}^{2}}{2}\\ T_{s} & 0\\ 0 & T_{s} \end{array}\right],$$

where *r*, *v*, θ, and (x,y) denote the position, speed, acceleration, and 2-D coordinates, respectively. *L* is the number of state elements which is composed of 2-D location and 2-D velocity, $T_{s}$ is the sampling time period, and *t* is the discrete-time step. The state is driven by the random process of $\theta_{t}$.

### 2.2. Measurement Model

The vector representation of the noisy measurements of the received signal strength (RSS) at *M* multi-sensors can be described as

(7) $$m_{t}=g\left(\psi_{t}\right)+\eta_{t},$$

where g(·) and $\eta_{t}$ are the observation function with respect to the state and additive measurement noise, respectively, and can be described in detail as follows.

(8) $$m_{t}={\left[m_{1,t},m_{2,t}\ldots m_{M,t}\right]}^{⊤},$$

(9) $$g\left(\psi_{t}\right)={\left[g_{1}\left(\psi_{t}\right),g_{2}\left(\psi_{t}\right)\ldots g_{M}\left(\psi_{t}\right)\right]}^{⊤},$$

(10) $$\eta_{t}={\left[\eta_{1,t},\eta_{2,t}\ldots \eta_{M,t}\right]}^{⊤}.$$

The RSS model is discussed in Reference [39], where the received power is expressed in dBm. Thus, the received signal power at the sensor *i* at the time step *t* is expressed as follows:

(11) $$m_{i,t}=g_{i}\left(\psi_{t}\right)+\eta_{i,t}=P_{0}-10\alpha \log_{10}\left(\frac{|l_{i}-r_{t}|}{d_{0}}\right)+\eta_{i,t},\;\mathrm{for}\;i=1,\cdots ,M,$$

where $P_{0}$ is the received power at the sensor from a reference distance $d_{0}$, $l_{i}$ is the position of the *i*-th sensor, $r_{t}={\left[r_{x,t},r_{y,t}\right]}^{⊤}$ is the location of the target at the time step *t*, α is an attenuation parameter that depends on the transmission medium, $\eta_{i,t}∼N$$\left(0,\sigma_{\eta_{i},t}^{2}\right)$ is a Gaussian noise process with the variance $\sigma_{\eta_{i},t}^{2}$, and *M* is the number of employed sensors in the networks. In this case, the likelihood function with respect to only $m_{i,t}$ becomes N$\left(m_{i,t};g_{i}\left(\psi_{t}^{}\right),\sigma_{\eta_{i},t}^{2}\right)$, which denotes a Gaussian distribution function at $m_{i,t}$ with the mean $g_{i}\left(\psi_{t}^{}\right)$ and the variance $\sigma_{\eta_{i},t}^{2}$, respectively.

## 3. Proposed Approach

In this section, we derive the minimax PF based on a fused manner of classical and Bayesian methods. This approach is derived by adopting the minimax strategy to standard PF (SPF). In the SPF, the risk function defined as the expected squared error is minimized, while the maximum risk function is minimized when the minimax strategy is adopted. The minimax strategy is reflected in the process of computing the weights of particles in the algorithm. Before we describe the minimax approach, we recall minimum mean square error estimator and PF first in order to derive the proposed approach smoothly. A Bayesian estimator that minimizes a predefined risk function can be described as follows.

(12) $$\min_{{\hat{\psi }}_{t}}R_{t}\left(\psi_{t},{\hat{\psi }}_{t}\right),$$

where, $\psi_{t}\in \Psi_{t}$, and the estimator ${\hat{\psi }}_{t}$ minimizes a risk function with respect to the random variable $\psi_{t}$. In the case of minimum mean square error (MMSE) estimator, the risk function can be described by

(13) $$R_{t}\left(\psi_{t},{\hat{\psi }}_{t}\right)=E\left(C_{t}\right),$$

where $E\left(\;\cdot \;\right)$ denotes the expected value, and the cost function $C_{t}\left(\;\cdot \;\right)$ is defined by the squared error if we let $\epsilon_{t}$ denotes the error of the estimator as follows.

(14) $$C_{t}=\epsilon_{t}^{2},\;\;\epsilon_{t}=\psi_{t}^{}-{\hat{\psi }}_{t}^{}.$$

The MMSE estimator that minimizes the mean square error defined as the risk function is described as follows:

(15) $$\begin{array}{ccc} {\hat{\psi }}_{t}^{\mathrm{MMSE}} & = & \min_{{\hat{\psi }}_{t}^{}}R_{t}^{\mathrm{MSE}}=\min_{{\hat{\psi }}_{t}^{}}\left[E\left(C_{t}\right)\right] \end{array}$$

(16) $$\begin{array}{cc} = & \int \psi_{t}p\left(\psi_{0:t}|m_{1:t}\right)d\psi_{t}, \end{array}$$

where $p\left(\psi_{0:t}|m_{1:t}\right)$ is the posterior density.

In PF, the problem is formulated as follows.

(17) $${\hat{\psi }}_{t}^{\mathrm{PF}}=\min_{{\hat{\psi }}_{t}^{}}R_{t}^{\mathrm{PF}}=\min_{{\hat{\psi }}_{t}^{}}\left[\sum_{\kappa =1}^{N}\left(w_{t}^{\kappa }C_{t}^{\kappa }\right)\right],$$

where $C_{t}^{\kappa }={\left(\psi_{t}^{\kappa }-{\hat{\psi }}_{t}\right)}^{2}$, $\epsilon_{t}^{\kappa }=\psi_{t}^{\kappa }-{\hat{\psi }}_{t}$, *N* is the number of employed particles, κ is the particle index, $w_{t}^{\kappa }$ is the weight of the particle κ at the time step *t*, and $\psi_{t}^{\kappa }$ is the particle with the index κ. Then, the MMSE estimate of PF can be derived as follows.

(18) $${\hat{\psi }}_{t}^{\mathrm{PF}}=\sum_{\kappa =1}^{N}w_{t}^{\kappa }\psi_{t}^{\kappa }.$$

In the problem of estimating a deterministic variable $\psi_{t}\in \Psi_{t}$ based on observation, an estimator ${\hat{\psi }}_{t}^{M}$ is the minimax estimator if its maximum risk is the minimum value by all possible estimators, which can be described as follows.

(19) $$\max_{\psi_{t}\in \Psi_{t}}R_{t}\left(\psi_{t},{\hat{\psi }}_{t}^{M}\right)=\min_{{\hat{\psi }}_{t}}\max_{\psi_{t}\in \Psi_{t}}R_{t}\left(\psi_{t},{\hat{\psi }}_{t}\right),$$

Therefore, the criterion of this classical estimator is avoiding the maximum risk by minimizing the maximum risk. To formulate the minimax strategy in PF framework, from Equations (13) and (14), we define a new minimax risk function as follows:

(20) $$\begin{array}{ccc} R_{t}^{M} & = & \max R_{t}^{\mathrm{PF}} \end{array}$$

(21) $$\begin{array}{cc} = & \max\sum_{\kappa =1}^{N}\left(w_{t}^{\kappa }C_{t}^{\kappa }\right) \end{array}$$

(22) $$\begin{array}{cc} = & \sum_{\kappa =1}^{N}\left(w_{t}^{M,\kappa }C_{t}^{\kappa }\right), \end{array}$$

where $w_{t}^{M,\kappa }$ denotes the modified weight associated with the particle index κ in minimax PF (MPF) because the minimax strategy is reflected when computing the weights of particles. Specifically, we select only one RSS-sensor-measurement among *M* that may incurs the highest risk. In other words, concerning *M* RSS-sensor-measurements, we use only one measurement that provides not the maximum weight but the minimum weight, which is highly counter-intuitive approach in terms of probabilistic point of view. In Equation (22), i.e., the risk function of MPF, only $w_{t}^{M,\kappa }$ is the factor of the magnitude of the risk because the particles are given, and we cannot modify them. When we associate the weight with only a single sensor-measurement, the weight is described as follows.

(23) $$w_{t}^{M,\kappa }\in {\left\{w_{i,t}^{\kappa }\right\}}_{i=1}^{M},$$

where the weight of the particle $\psi_{t}^{\kappa }$ can be computed in *M* ways, and $w_{i,t}^{\kappa }$ is the weight only based on the measurement $m_{i,t}$. Each particle is associated with the argument *i*, individually. Therefore, by associating the minimum weight among *M* weights for individual particle, we can maximize the risk function Equations (20)–(22), in minimax PF. If we consider the deterministic variable of classical minimax estimator as a random variable, we can modify the minimax Equation (19) with respect to MPF as follows.

(24) $$\begin{array}{ccc} {\hat{\psi }}_{t}^{M} & = & \min_{{\hat{\psi }}_{t}}R_{t}^{M} \end{array}$$

(25) $$\begin{array}{cc} = & \min_{{\hat{\psi }}_{t}}\max R_{t}^{\mathrm{PF}} \end{array}$$

(26) $$\begin{array}{cc} = & \min_{{\hat{\psi }}_{t}}\max_{{\left\{w_{i,t}^{\kappa }\right\}}_{\kappa =1}^{N}}R_{t}^{\mathrm{PF}}\left({\left\{w_{i,t}^{\kappa }\right\}}_{\kappa =1}^{N},{\hat{\psi }}_{t}\right) \end{array}$$

(27) $$\begin{array}{cc} = & \min_{{\hat{\psi }}_{t}^{}}\max_{{\left\{w_{i,t}^{\kappa }\right\}}_{\kappa =1}^{N}}\sum_{\kappa =1}^{N}\left(w_{i,t}^{\kappa }C_{t}^{\kappa }\right) \end{array}$$

(28) $$\begin{array}{cc} = & \min_{{\hat{\psi }}_{t}}\sum_{\kappa =1}^{N}\left(w_{t}^{M,\kappa }C_{t}^{\kappa }\right), \end{array}$$

where ${\left\{w_{t}^{M,\kappa }\right\}}_{\kappa =1}^{N}$ are defined as the weights in minimax PF that maximize the minimax risk function. If we let $w_{t}^{M,\kappa }≜w_{\tau ,t}^{\kappa }$, where

(29) $$\tau =\underset{i\in \left\{1,\ldots ,M\right\}}{\mathrm{argmin}}w_{i,t}^{\kappa },$$

the estimate of MPF at time step *t* is obtained from Equation (18) as follows.

(30) $$\begin{array}{ccc} {\hat{\psi }}_{t}^{M} & = & \sum_{\kappa =1}^{N}\omega_{t}^{M,\kappa }\psi_{t}^{\kappa }, \end{array}$$

(31) $$\begin{array}{cc} = & \sum_{\kappa =1}^{N}\omega_{\tau ,t}^{\kappa }\psi_{t}^{\kappa }. \end{array}$$

In this approach, we may select a different RSS-sensor-measurement that devalues the quality of the particle as low as possible for the computation of the weight of each particle. The minimax PF algorithm for the standard PF in wireless sensor networks is described in Algorithm 1 as follows:
**Algorithm 1:** The minimax PF algorithm for the standard PF in wireless sensor networks□ Initialization for κ=1,⋯,N, where *N* is the number of particles.   1. Random generation of initial particles:   $\psi_{0}^{\kappa }∼p\left(\psi_{0}\right)$, and assign initial weights: $\omega_{0}^{\kappa }=\frac{1}{N}$. end□ Sequential update for t=1,⋯,T, where *T* is the total time steps.  for κ=1,⋯,N,  2. Propagation of particles via a proposal density: (32)$$\psi_{t}^{\kappa }∼q\left(\psi_{t}^{}|\psi_{t-1}^{\kappa },m_{t}\right).$$  end  for κ=1,⋯,N,  3. Computing the weights of particles: (33)$$\omega_{i,t}^{\kappa }=p\left(m_{i,t}|\psi_{t}^{\kappa }\right)\;\mathrm{for}\;i=1,\ldots ,M$$   assuming the proposal density,   $q\left(\psi_{t}^{}|\psi_{t-1}^{\kappa },m_{t}\right)=p\left(\psi_{k}^{}|\psi_{t-1}^{\kappa }\right)$.  end  for κ=1,⋯,N,  4. Normalization. (34)$${\bar{\omega }}_{i,t}^{\kappa }=\frac{\omega_{i,t}^{\kappa }}{\sum_{\kappa =1}^{N}\omega_{i,t}^{\kappa }}\;\mathrm{for}\;i=1,\ldots ,M.$$  5. Selecting the minimum weight among *M* weights.$$\omega_{t}^{M,\kappa }={\bar{\omega }}_{\tau ,t}^{\kappa },\;\;\mathrm{where}\;\;\tau =\underset{i\in \left\{1,\ldots ,M\right\}}{\mathrm{argmin}}{\bar{\omega }}_{i,t}^{\kappa }.$$  end  6. Normalization of the weights: (35)$${\bar{\omega }}_{t}^{M,\kappa }=\frac{\omega_{t}^{M,\kappa }}{\sum_{\kappa =1}^{N}\omega_{t}^{M,\kappa }}\;\mathrm{for}\;\kappa =1,\cdots ,N.$$  7. Computing the estimate at the time step *t*: (36)$${\hat{\psi }}_{t}^{M}=\sum_{\kappa =1}^{N}{\bar{\omega }}_{t}^{M,\kappa }\psi_{t}^{\kappa }.$$  8. Resampling *N* particles.end

Therefore, as long as the computation of the weights of particles is performed with multiple measurements, this approach can be applied to any variants of PF. We only need to modify the step of computing the weights of particles in their algorithms to significantly mitigate the degeneracy of particles.

## 4. Performance Assessment

We evaluate the performance of the proposed MPFs, and compare with those of original PF variants, i.e., sequential-importance-resampling PF (SPF), regularized PF (RPF), auxiliary PF(APF), Kullback–Leibler-divergence PF (KLDPF), and Gaussian PF (GPF) for tracking a target in wireless sensor networks. We employ multi-sensors from two up to ten sensors in the experiments, and obtain various results depending on the number of employed sensors. We also compare the mean-risk based on Equation (20) and the variance of the weights of particles for both MPFs and original PF variants. The performance is assessed based on 300 runs of computer simulations, and 1000 particles (we do not improve the performance anymore beyond 1000) are used for all approaches. We consider two scenarios in terms of the target-motion-pattern: relatively a large or a small variance is considered for the state process noise based on Equation (1), where the larger the noise variance is, the larger the acceleration of the motion of the target becomes. The scenarios of large and small noises are denoted by $S_{L}$ and $S_{S}$, respectively. Therefore, the motion of the target is much greater under $S_{L}$ compared to the case under $S_{S}$ in a single time step, and particles undergo severe degeneracy under $S_{L}$. We denote the noise variances of the sate and the sensor-measurement, by $\sigma_{\theta }^{2},\sigma_{\eta }^{2}$, and $\Sigma^{2}=\left(\sigma_{\theta }^{2},\sigma_{\eta }^{2}\right)$, respectively. The noise variance for the state is assumed that $\sigma_{\theta }^{2}=\sigma_{\theta_{x}}^{2}=\sigma_{\theta_{y}}^{2}$. We used $\left(10^{-3},10^{-5}\right)$ and $\left(10^{-1},10^{-5}\right)$ for $\Sigma^{2}$, in $S_{S}$ and $S_{L}$, respectively. In the experiments of SPF, we used $(1,10^{-5})$ for $\Sigma^{2}$, in $S_{S}$. In the experiments of APF, we used $\left(10^{-3},10^{-3}\right)$ and $\left(10^{-1},10^{-3}\right)$ for $\Sigma^{2}$, in $S_{S}$ and $S_{L}$, respectively. The true initial state was generated with the expected values of ${\left[1,1,0.1,0.1\right]}^{⊤}$ within a uniform distribution, and the support-interval-length of [0.11] were used for $\left[S_{S}\;S_{L}\right]$, respectively. The initial particles were generated in the same way as the true state. The number of total time steps T=100. We performed 300 runs to obtain mean square error (MSE) of distance (MSED) and mean distance error (MDE) for each location over the *T* time steps. Cramér-Rao lower bound (CRLB) for the problem is provided in the Appendix A. In the simulations, we generated true trajectories based on the state model first and then deploy sensors uniformly in the area based on the starting and ending points of the target.

### 4.1. Standard Particle Filter

**Figure 1:**
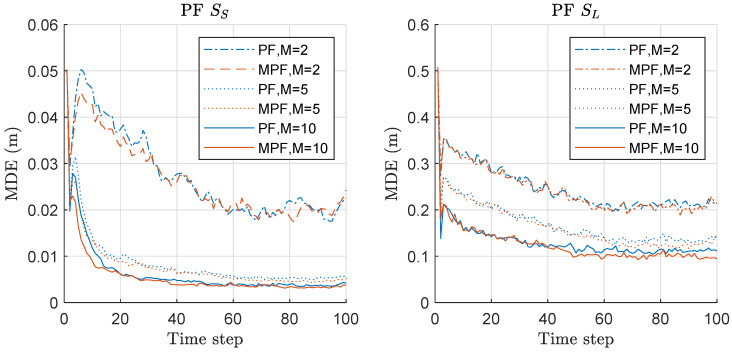
Mean distance error (MDE) performance comparison between particle filtering (PF) and minimax PF (MPF). Three hundred runs were performed with 1000 particles, where *M*, $S_{S}$, and $S_{L}$ denote the number of sensors, the scenario of the small state noise variance, and the scenario of the large state noise variance, respectively.

We begin with the sequential importance resampling particle filter as the standard particle filter (SPF) to compare the performance of the original and minimax versions. please add a and b for the subfigures and provide explanation in the caption. Mean distance error (MDE) of RPF and MRPF is compared in Figure 1. The left of Figure 1 shows the results under the scenario of $S_{S}$ with the various number of employed sensors *M* (i.e., 2, 5, and 10 sensors). When we use 2 sensors, both PF and MPF show similar MDE performance, while MPF outperforms PF when 5 and 10 sensors are employed. As *M* increases, the overall performances of both methods are improved. The right of Figure 1 shows the results under the scenario of $S_{L}$ with the various number of employed sensors *M*. The overall results under $S_{L}$ of both methods are worse than those under $S_{S}$ due to larger accelerations of the target. The superiority of MPF over PF is clear when M=5, while the superiority is not increasing significantly when 10 sensors are employed. When M=2, the performance difference between PF and MPF is not clear between the two methods, as in the case of $S_{S}$. Overall, MPF outperforms PF in terms of MDE as *M* increases, while the performance gain is not significantly increased beyond five sensors. The result shows that the minimax strategy is more effective under the $S_{L}$ scenario.

**Figure 2:**
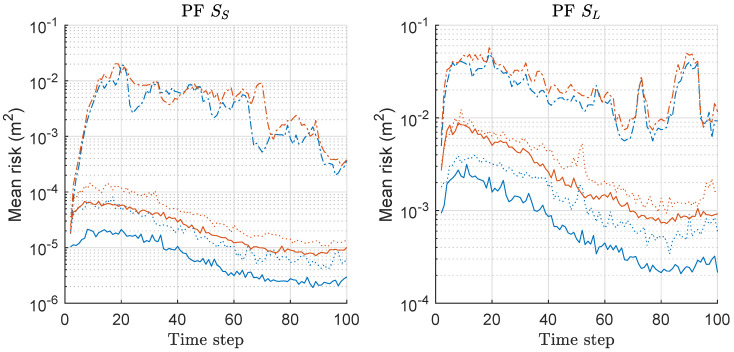
Mean-risk over 300 runs with 1000 particles based on Equation (20) for PF and MPF. Results regarding only $r_{x}$ are shown, and those regarding $r_{y}$ showed similar results. Legend: line style (dash-dot: M = 2; dotted: M = 5; solid: M = 10); (line color: blue: PF; red: MPF).

Based on Equation (20), we computed the risk function for both PF and MPF associated with the MDE-result of Figure 1, and the mean values over 300 runs are shown in Figure 2. The left and right of Figure 2 show the results under the scenarios of $S_{S}$ and $S_{L}$ with various *M*, respectively. The overall risk decreases as *M* increases regardless of the scenario. The gap of mean-risks between two methods is severer as *M* increases. The results show larger risks under $S_{L}$ compared to the case of $S_{S}$. The result of Figure 1 was obtained by minimizing the risk shown in Figure 2.

**Figure 3:**
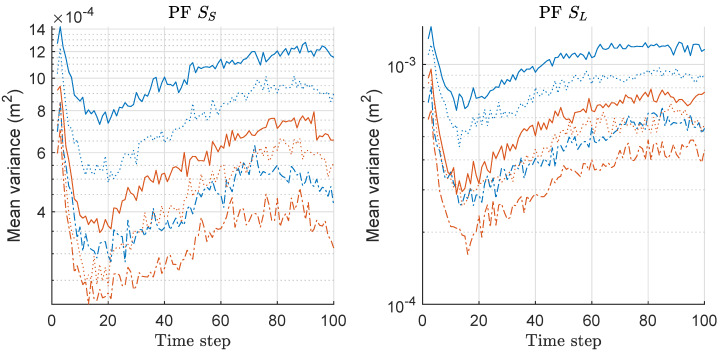
Mean-variance of the weights of particles over 300 runs with 1000 particles for PF and MPF. These results are associated with the results of MDE and mean-risk of Figure 1 and Figure 2. Legend: line style (dash-dot: M = 2; dotted: M = 5; solid: M = 10); (line color: blue: PF; red: MPF).

We computed the mean-variance of the weights of particles for both PF and MPF associated with the MDE/risk-results of Figure 1 and Figure 2, and the mean values over 300 runs are shown in Figure 3. The overall variances show similar patterns regardless of the scenario, and only depend on *M* while the variance-gap between two methods are severer as *M* increases; therefore, the minimax strategy is more effective as *M* increases, which is related to the MDE performance-result of Figure 1. In all cases, we obtained the significantly reduced variances of the weights of particles by MAPF compared to those of APF. We may keep increasing the size of *M* to obtain improved performance; however, the performance improvement may be saturated at a certain point with highly intensive computational complexity. Therefore, we confirmed that the proposed minimax strategy is highly effective in this problem with multi-RSS-measurements of sensor networks. Further, we verify the validity of the proposed strategy in more examples of variants of PFs in the following sections.

### 4.2. Auxiliary Particle Filter (APF)

Compared to SIRPF, the advantage of APF is that it naturally generates particles from the ones at the time step k−1 that are highly close to the true state [40]. In APF, the resampling is performed based on a certain particle estimate $\mu_{k}^{\kappa }$ that characterizes $p(\psi_{k}|\psi_{k-1}^{\kappa })$. When the process noise is very small, APF tends to be effective because $\mu_{k}^{\kappa }$ characterizes $p(\psi_{k}|\psi_{k-1}^{\kappa })$ well. Then, APF does not generate outliers often unlike SIRPF, and the weights are more even, which results in APF’s better performance than that of SIRPF. Therefore, APF can effectively show the outperforming result compared to SIRPF and is robust against the PI phenomenon due to the resampling procedure. APF returns to previous time steps of currently resampled particles, and then propagates the particles again for the current time step. The weights of the particles are computed based on the ratio of the likelihood functions of the resampled particles and newly propagated particles. We used the measurement noise variances $\sigma_{\eta }^{2}=10^{-3}$ instead of $10^{-5}$ in the experiments.

**Figure 4:**
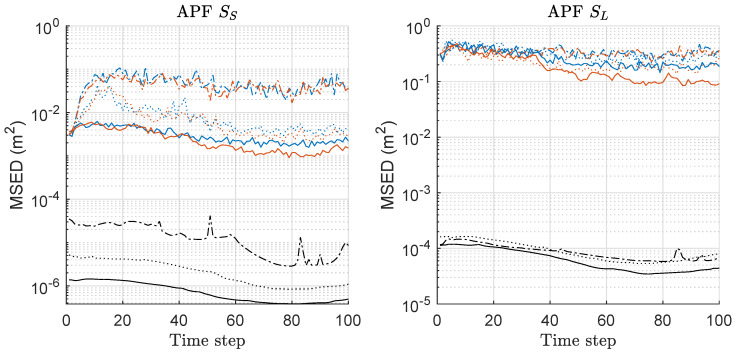
Mean square error of distance (MSED) performance comparison between auxiliary-PF (APF) and minimax APF (MAPF). Three hundred runs were performed with 1000 particles, where *M*, $S_{S}$, $S_{L}$ denote the number of sensors, the scenario of the small state noise variance, the scenario of the large state noise variance, respectively. Comparison with Cramér-Rao lower bound was also shown, as derived in the Appendix A. Legend: line style (dash-dot: M = 2; dotted: M = 5; solid: M = 10); (line color: blue: APF; red: MAPF; black: CRB).

MSED of APF, MAPF, and CRLB is compared in Figure 4. The left of Figure 4 shows the results under the scenario of $S_{S}$ with various *M*. Under the scenario $S_{S}$, MAPF outperforms APF with any *M*, and the MSED performance gap is much severer as *M* increases. Overall, as *M* increases, the performances of both methods are improved under $S_{S}$. The right of Figure 4 shows the results under the scenario of $S_{L}$ with various *M*. The overall performances for both methods are worse than those under $S_{S}$, and the outperforming results of MAPF over APF are similar to the case under $S_{S}$. Overall, MAPF outperforms APF in terms of the MSED performance, and the performance gap is severer as *M* increases and the minimax strategy is more effective, along with increasing *M*.

**Figure 5:**
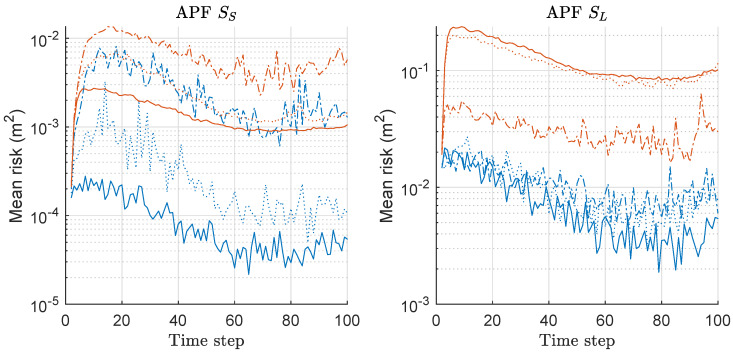
Mean-risk over 300 runs with 1000 particles based on Equation (20) for APF and MAPF. Results regarding only $r_{x}$ are shown, and those regarding $r_{y}$ showed similar results. Legend: line style (dash-dot: M = 2; dotted: M = 5; solid: M = 10); (line color: blue: APF; red: MAPF).

We computed the risk function based on Equation (20) for both APF and MAPF associated with the MSED-result of Figure 4, and the mean values over 300 runs are shown in Figure 5. The left and right of Figure 5 show the mean-risk results under the scenarios of $S_{S}$ and $S_{L}$ with various *M*, respectively. The overall risk is reduced as *M* increases regardless of the scenario. The gap of the risks between the two methods becomes severer as *M* increases. We obtained larger risks under $S_{L}$ compared to those under $S_{S}$.

**Figure 6:**
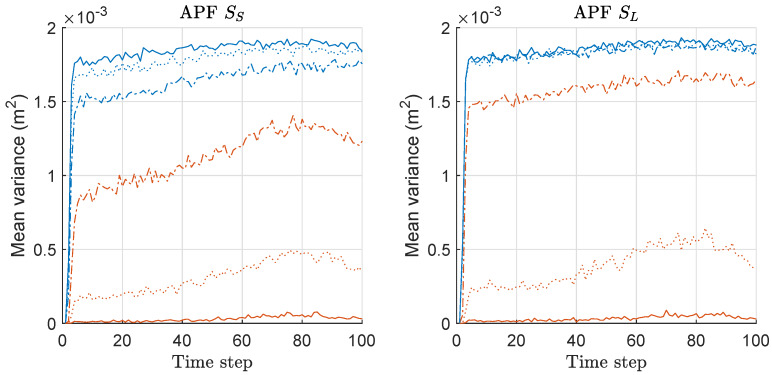
Mean-variance of the weights of particles over 300 runs with 1000 particles for APF and MAPF. These results are associated with the results of mean square error of distance (MSED) and mean-risk of Figure 4 and Figure 5. Legend: line style (dash-dot: M = 2; dotted: M = 5; solid: M = 10); (line color: blue: APF; red: MAPF).

We computed the mean-variance of the weights of particles for both APF and MAPF associated with the MSED/risk-results of Figure 4 and Figure 5, and the mean values over 300 runs are shown in Figure 6. For APF, the overall variance is similar regardless of the scenario and the size of *M*, whereas the variance of MAPF is significantly decreased as *M* increases. For both APF and MAPF, the variances do not depend on the scenario. Therefore, the variances gap between the two methods becomes severer as *M* increases. This means that the minimax strategy is more effective as the number of the sensor-measurements increases, which is related to the MSED performance results shown in Figure 4. The result of mean-variance is similar to that of PF/MPF shown in Figure 3, while the variance-gap between the two methods is more significant in the case of APF/MAPF than that in the case of PF/MPF. Therefore, the results show that the proposed minimax strategy is highly effective in the case of APF, as well, in this problem with multi-RSS-measurements of sensor networks.

### 4.3. Regularized PF (RPF)

PI phenomenon is another defect of the SPF besides the degeneracy issue in the practical implementations. The SPF is modified to resolve the PI problem in the RPF algorithm [41]. A kernel density that perturbs the state of particles is employed to develop the diversity of the states of the particles. In RPF, the posterior density is approximated by a rescaled kernel density.

**Figure 7:**
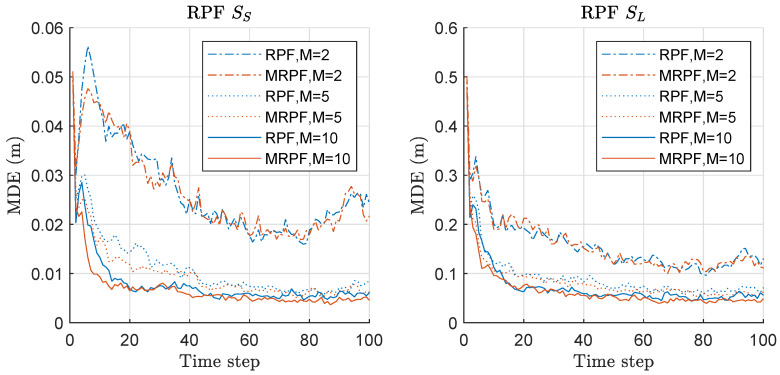
Mean distance error (MDE) performance comparison between regularized PF (RPF) and minimax RPF (MRPF). Three hundred runs were performed with 1000 particles, where *M*, $S_{S}$, and $S_{L}$ denote the number of sensors, the scenario of the small state noise variance, and the scenario of the large state noise variance, respectively.

Mean distance error (MDE) of RPF and MRPF is compared in Figure 7. We assess the MDE performance instead of MSED because we could see the result more clearly with MDE rather than MSED in this case of RPF/MRPF. The left of Figure 7 shows the results under the scenario of $S_{S}$ with various *M*. Under the $S_{S}$ scenario, MRPF outperforms RPF when M=5,10, while the performance is similar when M=2. Overall, as *M* increases, the performances of both methods are improved. The right of Figure 7 shows the results under the scenario of $S_{L}$ with various *M*. The overall performance is worse than that under the $S_{S}$ scenario, and the performances of the two methods are similar when M=2 under both scenarios. Overall, MRPF outperforms RPF in terms of MDE, and the performance gap becomes severer as *M* increases, and the minimax strategy becomes more effective.

**Figure 8:**
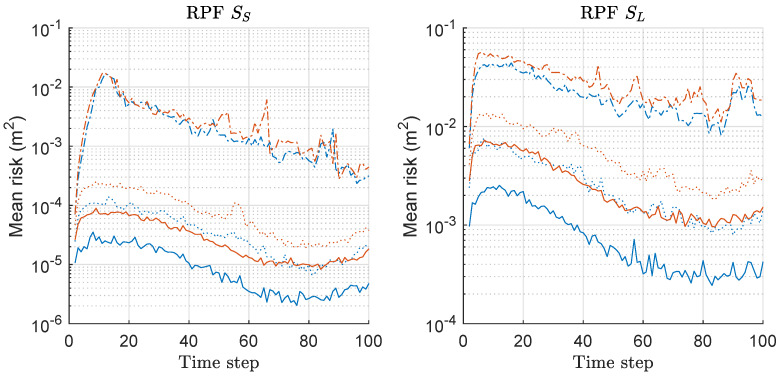
Mean-risk over 300 runs with 1000 particles based on Equation (20) for RPF and MRPF. Results regarding only $r_{x}$ are shown, and those regarding $r_{y}$ showed similar results. Legend: line style (dash-dot: M = 2; dotted: M = 5; solid: M = 10); (line color: blue: RPF; red: MRPF).

We computed, the risk function based on Equation (20) for both RPF and MRPF associated with the MDE-result of Figure 7, and the mean values over 300 runs are shown in Figure 8. The left and right of Figure 8 show the mean-risk results under the scenarios of $S_{S}$ and $S_{L}$ with various *M*, respectively. The overall risk is reduced as *M* increases regardless of the scenario as in the case of previous results of PFs. The gap of the mean-risks between the two methods becomes severer as *M* increases. We have larger risks under $S_{L}$ compared to the case of $S_{S}$. The mean-risk result of RPF/MRPF is similar to that of PF/MPF.

**Figure 9:**
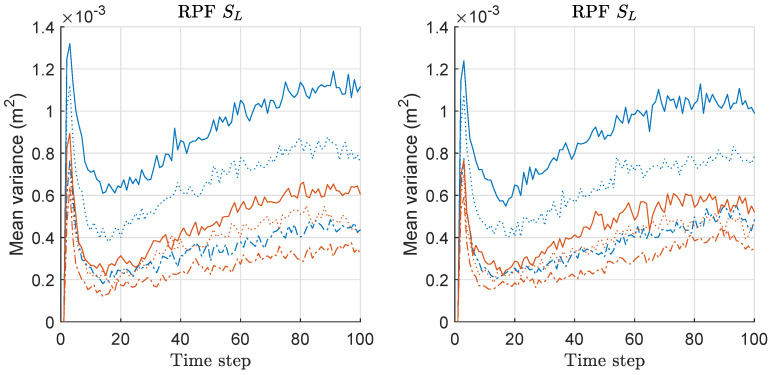
Mean-variance of the weights of particles over 300 runs with 1000 particles for RPF and MRPF. These results are associated with the results of MDE and mean-risk of Figure 7 and Figure 8. Legend: line style (dash-dot: M = 2; dotted: M = 5; solid: M = 10); (line color: blue: RPF; red: MRPF).

We computed the mean-variance of the weights of particles for both RPF and MRPF associated with the MDE/risk-results of Figure 7 and Figure 8, and the mean values over 300 runs are shown in Figure 9. For RPF, the variance of RPF increases along with the increased *M*, whereas that of MRPF remains similarly regardless of the increased *M*; therefore, the gap of variance difference is increased along with the increased *M* regardless of the scenario. This means that the minimax strategy is more effective as *M* increases, which is related to the MDE performance result of Figure 7. The result of mean-variance is similar to the case of PF/MPF as shown in Figure 3. Therefore, we also confirmed that the proposed minimax strategy is highly effective in the case of RPF with multiple measurements of sensor networks.

### 4.4. Kullback-Leibler Divergence PF (KLDPF)

In KLDPF, the number of employed particles is optimized based on a predetermined error bound at each time step [23]. The number of employed particles is adjusted to reduce redundant particles and also unnecessary computations, accordingly, while the performance is not affected. In this assessment, we apply the error bound of 0.001, the initial number of employed particles is 500 and the maximum number of particles is bounded by 1000; the probability bound is 0.01, the bin size is $1/2\times \sqrt{\sigma_{\theta_{}}}\times 4\left(L\right)$; 1/2 is determined from $T_{s}/2$ in E. Although KLDPF adaptively optimizes the number of particles at every time step, the algorithm inherently requires a higher computational cost than those of other PF variants.

**Figure 10:**
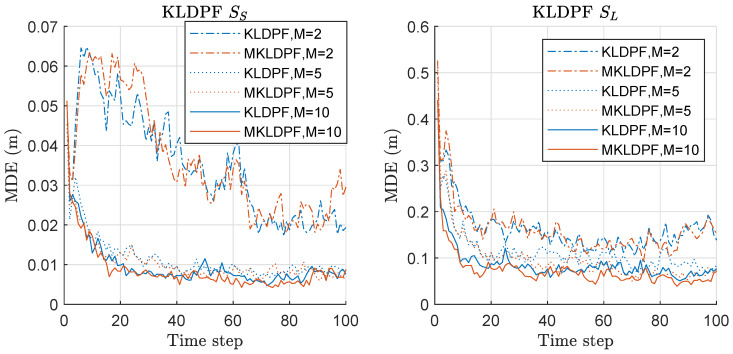
Mean distance error (MDE) performance comparison between Kullback–Leibler divergence-PF (KLDPF) and minimax KLDPF (MKLDPF). Three hundred runs were performed with 1000 particles, where *M*, $S_{S}$, and $S_{L}$ denote the number of sensors, the scenario of the small state noise variance, and the scenario of the large state noise variance, respectively.

MDE of KLDPF and MKLDPF is compared in Figure 10. The left of Figure 10 shows the results under the scenario of $S_{S}$ with various *M*. Under the $S_{S}$ scenario, MKLDPF outperforms KLDPF when *M* is equal to 5 or larger, while the performance is similar to each other when M=2. Overall, MKLDPF outperforms KLDPF when *M* is increased above 2. The right of Figure 10 shows the results under the scenario of $S_{L}$ with various *M*. The overall results are worse than those under $S_{S}$ for both methods. The superiority of MKLDPF over KLDPF is clear when M=5,10. When M=2 under $S_{L}$, the performance difference between KLDPF and MKLDPF is not clear as in the case of $S_{S}$. Overall, MKLDPF outperforms KLDPF in terms of MDE, and the performance gap is severer and the minimax strategy is more effective as *M* increases. The pattern of the MDE result of KLDPF/MKLDPF shown in Figure 10 is highly similar to that of RPF/MRPF shown in Figure 7.

**Figure 11:**
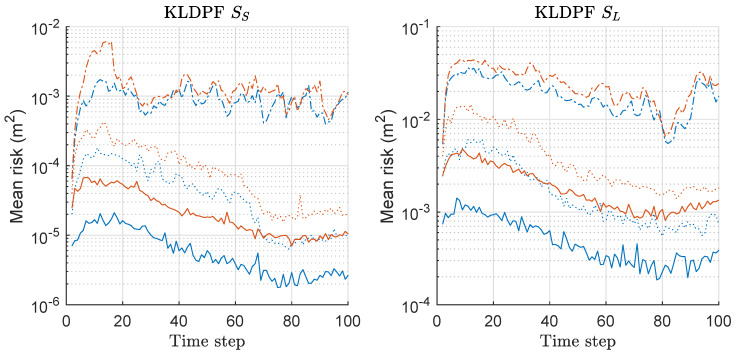
Mean-risk over 300 runs with 1000 particles based on Equation (20) for KLDPF and MKLDPF. Results regarding only $r_{x}$ are shown, and those regarding $r_{y}$ showed similar results. Legend: line style (dash-dot: M = 2; dotted: M = 5; solid: M = 10); (line color: blue: KLDPF; red: MKLDPF).

We computed the risk function based on Equation (20) for both KLDPF and MKLDPF associated with the MDE-result of Figure 10, and the mean values over 300 runs are shown in Figure 11. The left and right of Figure 11 show the mean-risk results under the scenarios of $S_{S}$ and $S_{L}$ with various *M*, respectively. The overall risk decreases as *M* increases regardless of the scenario. The gap of the risks between the two methods is severer as *M* increases. We obtain larger risks under $S_{L}$ compared to the case of $S_{S}$ regardless of the method and *M*. The MDE-result of Figure 10 was obtained by minimizing the risk in Figure 11. The pattern of the mean-risk-result shown in Figure 11 is also highly similar to that of RPF/MRPF shown in Figure 8.

**Figure 12:**
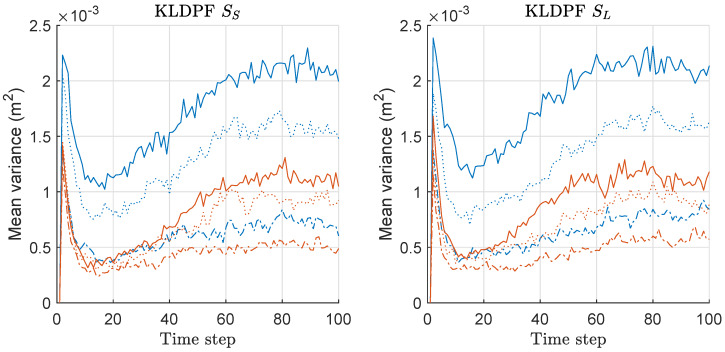
Mean-variance of the weights of particles over 300 runs with 1000 particles for KLDPF and MKLDPF. These results are associated with the results of MDE and mean-risk of Figure 10 and Figure 11. Legend: line style (dash-dot: M = 2; dotted: M = 5; solid: M = 10); (line color: blue: KLDPF; red: MKLDPF).

We computed the mean-variance of the weights of particles for both KLDPF and MKLDPF associated with the MDE/risk-results of Figure 10 and Figure 11, and the mean values over 300 runs are shown in Figure 12. The gap of the variances between the two methods becomes severer as *M* increases regardless of the scenario. This means that the minimax strategy is more effective as *M* increases, which is closely related to the MDE performance result of Figure 10. In all cases, we obtained the significantly reduced variance of the weights of particles by the minimax strategy as shown in Figure 12. The pattern of all the results of MDE-performance, mean-risk, mean-variance of KLDPF/MKLDPF are highly similar to that of RPF/MRPF, while the computational complexities of RPF and KLDPF are quite different, as we show in the result of it shortly. Therefore, we confirmed that the proposed minimax strategy is highly effective in the case of KLDPF, as well.

### 4.5. Gaussian PF (GPF)

In the GPF algorithm, an additional particle generation step is required prior to the particle propagation step. That is, particles are generated based on a Gaussian distribution where the mean value and the covariance are adopted from the previous estimate $\left({\hat{\psi }}_{k-1}^{}\right)$ and the weighted sample covariance, $C_{k-1}=\sum_{\kappa =1}^{N}\omega_{k-1}^{\kappa }\left(\psi_{k-1}^{\kappa }-{\hat{\psi }}_{k-1}\right){\left(\psi_{k-1}^{\kappa }-{\hat{\psi }}_{k-1}\right)}^{⊤}$. Then, particles are propagated by the prior density. There is no resampling process in the GPF algorithm, which significantly reduces its computational complexity. For details about GPF, refer to Reference [26].

**Figure 13:**
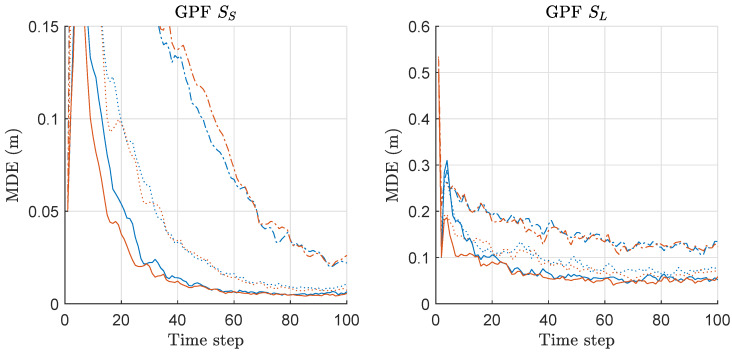
Mean distance error (MDE) performance comparison between Gaussian-PF (GPF) and minimax GPF (MGPF). Three hundred runs were performed with 1000 particles, where *M*, $S_{S}$, $S_{L}$ denote the number of sensors, the scenario of the small state noise variance, the scenario of the large state noise variance, respectively. Legend: line style (dash-dot: M = 2; dotted: M = 5; solid: M = 10); (line color: blue: GPF; red: MGPF).

MDE of GPF and MGPF is compared in Figure 13. The left and right of Figure 13 show the results under the scenarios of $S_{S}$ and $S_{L}$ with various *M*, respectively. Overall, MGPF slightly outperforms GPF when *M* is increased above 2. The overall results under $S_{L}$ are worse than those under $S_{S}$. The superiority of MGPF over GPF is not as much as that of the other PF variants that have been investigated so far. Nonetheless, overall, MGPF outperforms GPF in terms of MDE performance when we increase the number of employed sensors. It needs to be noted that GPF does not require the resampling process that also incurs the side effect, such as particle impoverishment. Therefore, the minimax strategy is not as effective as the cases of the other PF variants.

**Figure 14:**
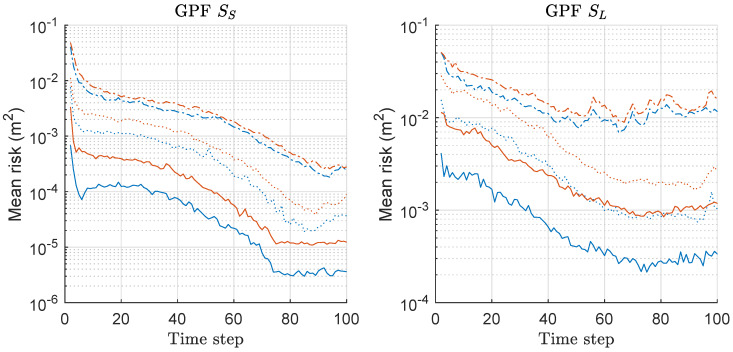
Mean-risk over 300 runs with 1000 particles based on Equation (20) for GPF and MGPF. Results regarding only $r_{x}$ are shown, and those regarding $r_{y}$ showed similar results. Legend: line style (dash-dot: M = 2; dotted: M = 5; solid: M = 10); (line color: blue: GPF; red: MGPF).

We computed the risk function based on Equation (20) for both GPF and MGPF associated with the MDE-result of Figure 13, and the mean values over 300 runs are shown in Figure 14.

The left and right of Figure 14 show the mean-risk results under the scenarios of $S_{S}$ and $S_{L}$ with various *M*, respectively. The overall risk decreases as *M* increases, regardless of the scenario, like the previously shown results of the other PFs. The relative gap of the risks between the two methods becomes severer as *M* increases for both scenarios. Overall, we have larger risks under $S_{L}$ compared to the case of $S_{S}$;

We also note that the relative gap of the mean-risks is the severest when M=10 under $S_{L}$ among all results in Figure 14.

**Figure 15:**
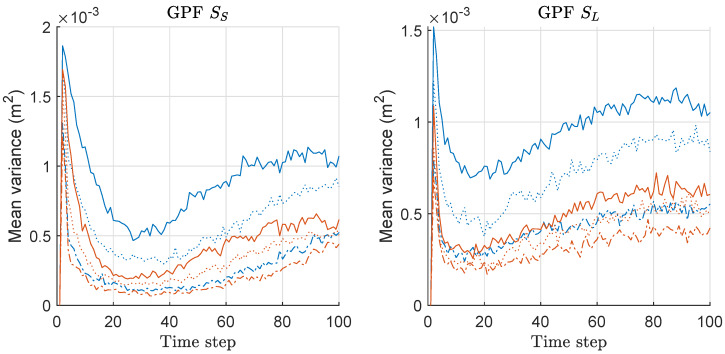
Mean-variance of the weights of particles over 300 runs with 1000 particles for GPF and MGPF. These results are associated with the results of MDE and mean-risk of Figure 13 and Figure 14. Legend: line style (dash-dot: M = 2; dotted: M = 5; solid: M = 10); (line color: blue: GPF; red: MGPF).

We computed the mean-variance of the weights of particles for both GPF and MGPF associated with the MDE/risk-results of Figure 13 and Figure 14, and the mean values over 300 runs are shown in Figure 15. The gap of the variances between two methods becomes severer as *M* increases in both scenarios, and the relative gap is slightly severer under $S_{L}$ compared to the case under $S_{S}$. This means that the minimax strategy is more effective as *M* and $\sigma_{\theta }^{2}$ increases. The pattern of the overall variance-results of GPF and MGPF shown in Figure 15 is highly similar to those of RPF/MRPF and KLDPF/MKLDPF shown in Figure 9 and Figure 12. The pattern of all results of MDE performance, mean-risk, and mean-variance of GPF/MGPF is highly similar to those of RPF/MRPF and KLDPF/MKLDPF. Therefore, we confirmed that the proposed minimax strategy is effective in the case of GPF, as well, although the effect is not as much as that in the other PF variants.

### 4.6. Processing Time

**Figure 16:**
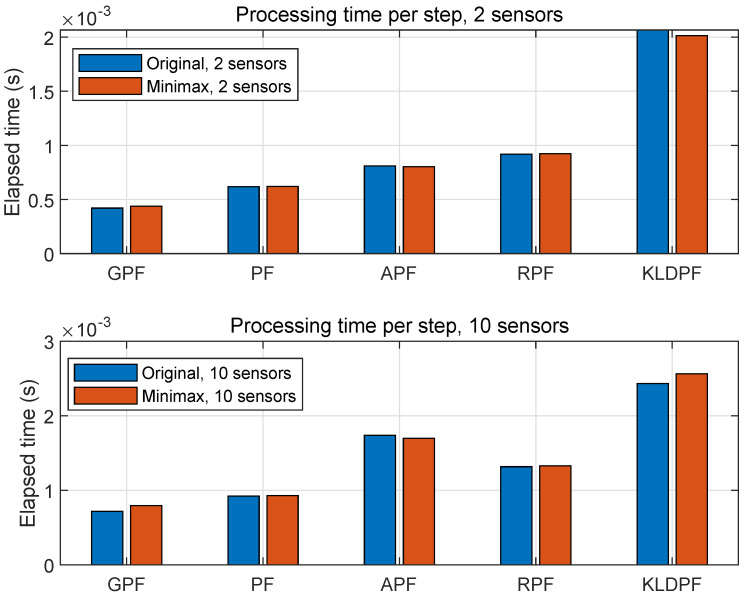
Mean-elapsed time for the one time step over 300 runs with 1000 particles for all PFs. Note that there is no resampling process required for GPF.

For all methods, we measured the processing time in terms of the average-elapsed-time for the one time step out of *T* during the MATLAB simulations, and the result is shown in Figure 16. The top and bottom figures are concerning two and ten sensors, respectively, in the networks. The elapsed time concerning ten sensors is not five times longer than the case of two sensors but elapsed approximately between 1.5 and 2 times longer. As clearly seen, KLDPF requires the most duration of elapsed time among all the PFs. GPF and MGPF require the least duration of elapsed time because GPF does not require the resampling process. There is no significant time difference between minimax and original PF variants. The minimax strategy affects the processing time in terms of the computation of finding the minimum value of the weights among *M*, while original PF variants require the computation of joint probability density function of *M* densities; therefore, overall computational complexities of two frameworks do not show significant time-difference. The minimax PFs do not require the computation of the joint probability density, which can be intractable sometimes.

### 4.7. Discussion

In all PF variants, the minimax versions showed better tracking performance than original PF variants in terms of MSED/MDE, while both frameworks showed similar processing-time-performance during the MATLAB experiments. The experimental simulation results can be summarized as follows. Concerning MSED/MDE performance, overall: both original and minimax PFs showed similar performances in terms of MSED/MDE with two sensors, while minimax PFs clearly outperformed original PFs with sensors over two. Besides, the performance gap is slightly more evident under $S_{L}$ compared to that under $S_{S}$ due to the severer degeneracy problem under $S_{L}$. Nevertheless, the performance gap was not significantly different between the results with five sensors and those with ten sensors. Concerning the mean-risk, overall, the risk was the lowest with the maximum number of sensors under $S_{S}$, while the difference gap between two PF-frameworks is relatively the largest with the maximum number of sensors regardless of the scenario. When two sensors are employed, unlike the case of the MSED/MDE-performance, the mean-risk of the minimax PFs is clearly higher than that of original PF variants. Concerning the mean-variance of the weights of particles, the results only depend on the number of employed sensors regardless of the scenario, and the gap of the mean-variance is severer as *M* increases due to the effect of minimax strategy that results in the improved tracking performance.

## 5. Conclusions

In this paper, we proposed the minimax PF for the problem of tracking a target based on RSS measurements in wireless sensor networks where a large number of sensor-measurements are available. We verified the validity of the proposed minimax strategy in various scenarios in terms of *M* (the number of sensors) and $\sigma_{\theta }^{2}$ (the variance of the state noise) by applying to various variants of PF. The adopted minimax strategy is more effective when we employ a large number of the sensors in the networks compared to the case of a small number of the sensors employed; therefore, we obtained the significantly reduced variance of the weights of particles that derives the robustness against the degeneracy problem of generic PF. The robustness against the degeneracy resulted in the improved tracking performance beyond the asymptotically optimal performance of the original PF variants. Furthermore, the strategy is more effective when the dynamic state-space model varies with a larger variance, where the particles undergo a severer degeneracy problem. It needs to be noted that the minimax strategy is effective on condition that the signal-to-noise ratio is above a certain value that is good enough for tracking. Otherwise, MPFs may not outperform regular PFs.

## Appendix A. Derivation of Cramér-Rao Lower Bound

The variance of any unbiased estimate, $\hat{\psi }$ is bounded as follows:

(A1) $$\mathrm{Var}\left({\hat{\psi }}_{e}\right)\geq {\left[I^{-1}\left(\psi \right)\right]}_{ee},\;\mathrm{for}\;e=1,\ldots ,L,$$

where I is the Fisher information matrix, ψ is a vector state, *e* is the element index, and *L* is the dimension of the state vector. When the measurement noise is Gaussian and the measurement is given by

(A2) $$m∼N\left(g\left(\psi \right),C_{\eta }\left(\psi \right)\right),$$

we can obtain the following, for e,l=1,…,L,

(A3) $$\begin{array}{cc} {\left[I(\psi )\right]}_{el}= & {\left[\frac{\partial g(\psi )}{\partial \psi_{e}}\right]}^{⊤}C_{\eta }^{-1}\left(\psi \right)\left[\frac{\partial g(\psi )}{\partial \psi_{l}}\right]+\\ & \frac{1}{2}\mathrm{tr}\left[\left(C_{\eta }^{-1}\left(\psi \right)\frac{\partial C_{\eta }\left(\psi \right)}{\partial \psi_{e}}C_{\eta }^{-1}\left(\psi \right)\frac{\partial C_{\eta }\left(\psi \right)}{\partial \psi_{l}}\right)\right]\; \end{array}$$

where

(A4) $$\frac{\partial g(\psi )}{\partial \psi_{i}}={\left[\frac{\partial {\left[g(\psi )\right]}_{1}}{\partial \psi_{i}},\frac{\partial {\left[g(\psi )\right]}_{2}}{\partial \psi_{i}},\cdots ,\frac{\partial {\left[g(\psi )\right]}_{M}}{\partial \psi_{i}}\right]}^{⊤},$$

(A5) $$\frac{\partial C_{\eta }\left(\psi \right)}{\partial \psi_{i}}=\left[\begin{array}{cccc} \frac{\partial {\left[C_{\eta }\left(\psi \right)\right]}_{11}}{\partial \psi_{i}} & \frac{\partial {\left[C_{\eta }\left(\psi \right)\right]}_{12}}{\partial \psi_{i}} & \cdots & \frac{\partial {\left[C_{\eta }\left(\psi \right)\right]}_{1M}}{\partial \psi_{i}}\\ \frac{\partial {\left[C_{\eta }\left(\psi \right)\right]}_{21}}{\partial \psi_{i}} & \frac{\partial {\left[C_{\eta }\left(\psi \right)\right]}_{22}}{\partial \psi_{i}} & \cdots & \frac{\partial {\left[C_{\eta }\left(\psi \right)\right]}_{2M}}{\partial \psi_{i}}\\ \vdots & \vdots & \ddots & \vdots \\ \frac{\partial {\left[C_{\eta }\left(\psi \right)\right]}_{M1}}{\partial \psi_{i}} & \frac{\partial {\left[C_{\eta }\left(\psi \right)\right]}_{M2}}{\partial \psi_{i}} & \cdots & \frac{\partial {\left[C_{\eta }\left(\psi \right)\right]}_{MM}}{\partial \psi_{i}} \end{array}\right],$$

$g\left(\psi \right)={\left[g_{1}\;\cdots \;g_{M}\right]}^{⊤}$, and the covariance matrix $C_{\eta }\left(\psi \right)=\mathrm{diag}\left(\sigma_{\eta_{1}}^{2}\;\cdots \;\sigma_{\eta_{M}}^{2}\right)$, where diag $\left(\;\cdot \;\right)$ denotes a diagonal matrix, and the variance (power) of the measurement noise $\eta_{i}$:

(A6) $$\sigma_{\eta_{i}}^{2}={g_{i}}^{2}\;\cdot \;10^{\left(-{\mathrm{SNR}}_{m_{i}}/10\right)},$$

where SNR$m_{i}$ is the signal-to-noise ratio (SNR) in dB for the corresponding $m_{i}$, and are computed as follows:

(A7) $${\mathrm{SNR}}_{m_{i}}=10\log_{10}\left[\frac{{g_{i}}^{2}}{\sigma_{\eta_{i}}^{2}}\right].$$

The Fisher information is a 4×4 matrix, and ${\left[I(\psi )\right]}_{11}$ and ${\left[I(\psi )\right]}_{22}$ are the corresponding elements for $r_{x}$ and $r_{y}$, respectively. Therefore,

(A8) $$\mathrm{Var}\left({\hat{r}}_{x}\right)\geq {\left[I^{-1}\left(\psi \right)\right]}_{11},\;\;\;\mathrm{Var}\left({\hat{r}}_{y}\right)\geq {\left[I^{-1}\left(\psi \right)\right]}_{22}.$$

If CRLB for the distance estimation, i.e., $\sqrt{r_{x}^{2}+r_{y}^{2}}$ is to be computed, we can use vector parameter CRLB for transformations, and is easily derived as follows [42]. If we define $\alpha =h\left(\psi \right)=\sqrt{r_{x}^{2}+r_{y}^{2}}$, CRLB is derived as

(A9) $$\mathrm{Var}\left(\hat{\alpha }\right)\geq \frac{\partial h(\psi )}{\partial \psi }I^{-1}\left(\psi \right)\frac{\partial h{\left(\psi \right)}^{⊤}}{\partial \psi },$$

where a Jacobian matrix $\frac{\partial h(\psi )}{\partial \psi }$ is described as

(A10) $$\frac{\partial h(\psi )}{\partial \psi }=\left[\begin{array}{cccc} \frac{\partial h(\psi )}{\partial \psi_{1}} & \frac{\partial h(\psi )}{\partial \psi_{2}} & \cdots & \frac{\partial h(\psi )}{\partial \psi_{L}} \end{array}\right].$$
